# Genomewide Analysis of Mode of Action of the *S*-Adenosylmethionine Analogue Sinefungin in Leishmania infantum

**DOI:** 10.1128/mSystems.00416-19

**Published:** 2019-10-15

**Authors:** Arijit Bhattacharya, Mansi Sharma, Charles Pakkinathan, Barry P. Rosen, Philippe Leprohon, Marc Ouellette

**Affiliations:** a Division of Infectious Disease and Immunity, CHU de Quebec Research Center, Quebec, Quebec, Canada; b Department of Microbiology, Infectious Disease and Immunology, University Laval, Quebec, Quebec, Canada; c Department of Cellular Biology and Pharmacology, Herbert Wertheim College of Medicine, Florida International University, Miami, Florida, USA; University of Illinois at Chicago

**Keywords:** *Leishmania*, single-nucleotide variants, copy number variation, transporter, methyltransferase, *S*-adenosylmethionine, drug resistance mechanisms

## Abstract

To further our understanding of one-carbon metabolism in the protozoan parasite *Leishmania*, we conducted genomic screens to study how the parasite responded to sinefungin (SNF) selection. SNF is a structural analogue of *S*-adenosylmethionine (AdoMet), a key methyl group donor to a number of biomolecules. One screen consisted of sequencing SNF-resistant mutants generated by stepwise selection with gradually increasing drug concentrations. These studies demonstrated deletion of the AdoMet transporter (AdoMetT1) by intergenic recombination as a crucial loss-of-function marker for SNF resistance. The second screen consisted of Cos-seq, a gain-of-function cosmid-based genomewide functional screen with increasing SNF concentration coupled to next-generation sequencing. Cosmids enriched in that screen and sequenced led to the identification of (i) the AdoMet synthetase (METK) as the major SNF target, (ii) an mRNA [(guanine-N7)-methyltransferase (CMT1)], (iii) a leucine carboxyl methyltransferase (LCMT), (iv) two tryparedoxin genes, and (v) two protein phosphatase regulatory genes. Further functional exploration indicated that LCMT interacts with one phosphatase catalytic subunit (PP2AC) and that mutation of the C-terminal leucine residue of PP2AC affects sinefungin susceptibility. These holistic screens led to the identification of transporters, biosynthetic genes, RNA and protein methyltransferases, as well as phosphatases linked to AdoMet-mediated functions in *Leishmania*.

**IMPORTANCE** The two main cellular metabolic one-carbon donors are reduced folates and *S*-adenosylmethionine, whose biosynthetic pathways have proven highly effective in chemotherapeutic interventions in various cell types. Sinefungin, a nucleoside analogue of *S*-adenosylmethionine, was shown to have potent activity against the protozoan parasite *Leishmania*. Here, we studied resistance to sinefungin using whole-genome approaches as a way to further our understanding of the role of *S*-adenosylmethionine in this parasite and to reveal novel potential drug targets. These approaches allowed the characterization of novel features related to *S*-adenosylmethionine function in *Leishmania* which could further help in the development of sinefungin-like compounds against this pathogenic parasite.

## Abstract (precis)

The two main cellular metabolic one-carbon donors are reduced folates and *S*-adenosylmethionine, whose biosynthetic pathways have proven highly effective in chemotherapeutic interventions in various cell types. Sinefungin, a nucleoside analogue of *S*-adenosylmethionine, was shown to have potent activity against the protozoan parasite *Leishmania*. Here, we studied resistance to sinefungin using whole-genome approaches as a way to further our understanding of the role of *S*-adenosylmethionine in this parasite and to reveal novel potential drug targets. These approaches allowed the characterization of novel features related to *S*-adenosylmethionine function in *Leishmania* which could further help in the development of sinefungin-like compounds against this pathogenic parasite.

## INTRODUCTION

Parasites of the genus *Leishmania* cause a range of devastating and often fatal diseases in humans and domestic animals and affect an estimated 700,000 to 1 million people each year (1). Treatment of leishmaniasis relies primarily on chemotherapy with four drugs, namely, pentavalent antimonials, miltefosine, amphotericin B, and paromomycin. None are ideal, and alternatives are urgently needed (2).

The two main cellular metabolic one-carbon donors are reduced folates and *S*-adenosylmethionine (AdoMet), and inhibitors against biosynthetic genes involved in these one-carbon donor pathways have proven highly effective in chemotherapeutic interventions in various cell types. Our understanding of folate metabolism in *Leishmania* has emerged mostly from studies of parasites selected for resistance to the model drug methotrexate (MTX) (3, 4). Our understanding of AdoMet metabolism in *Leishmania* is less advanced, but a link between AdoMet and folate metabolism has been established in *Leishmania*. Indeed, cells resistant to MTX overexpressed *S*-adenosylmethionine synthetase (MetK) (5), the key enzyme in AdoMet biosynthesis, and transfection of MetK in *Leishmania* facilitates the emergence of high-level resistance to MTX (6).

AdoMet is involved in the methylation of lipids, proteins, and nucleic acids and is a donor of propylamine groups or methylene groups for a number of molecules, including polyamines, fatty acids, or biotin (7, 8). AdoMet, through the trans-sulfuration pathway, can be metabolized into cysteine and glutathione (GSH) and in the spermidine-glutathione conjugate trypanothione (TSH) in *Leishmania* (9). Overexpression of MetK was observed in antimony-resistant *Leishmania*, most likely because of this trans-sulfuration pathway (10). Overexpression of MetK has also been associated with other drugs in *Leishmania*, including allopurinol (11), and with the AdoMet analogue sinefungin (SNF) (12).

SNF, a nucleoside analogue of AdoMet, was reported to have potent antileishmanial activity both in cell culture and animals models (13–15). SNF was shown to use the same uptake system as AdoMet (16), and the AdoMet-SNF transporter of *Leishmania* (AdoMetT1) (17) is a member of the folate-biopterin-transporter (FBT) family (18).

Similarly to MTX resistance studies that led to an understanding of folate metabolism in *Leishmania*, we hypothesized that studying resistance to SNF may lead to further understanding of the role of AdoMet in this parasite and may lead to potential drug targets. Sequencing of resistant parasites is now well established as a technique for gaining insight into resistance mechanisms and modes of action of drugs (19). In addition to genome sequencing, genomewide gain-of-function screens exploiting cosmid-based functional screening coupled to next-generation sequencing (Cos-seq) has expedited the discovery of drug resistance/target genes in *Leishmania* (20–22). In this study, we combined the sequencing of SNF-resistant mutants with a Cos-seq screen selecting for resistance to SNF for an increased understanding of AdoMet metabolism in *Leishmania*. These holistic screens led to the identification of transporters, biosynthetic genes, RNA and protein methyltransferases as well as phosphatases linked to AdoMet mediated functions in *Leishmania*.

## RESULTS

### Generation of sinefungin-resistant mutants by stepwise selection.

**FIG 1:**
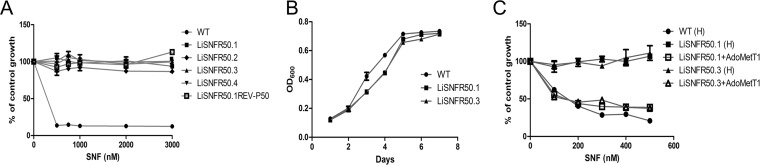
Properties of L. infantum SNF-resistant mutants. (A) Drug response analysis of wild-type (WT) *Leishmania* (●) and SNF-resistant mutants LiSNFR50.1 (■), LiSNFR50.2 (♦), LiSNFR50.3 (▲), LiSNFR50.4 (▼), and LiSNFR50.1 grown for 50 passages without SNF (small white box on black background). Data are means ± standard errors of the means (SEM) (error bars) from at least three independent experiments. (B) L. infantum SNF-resistant mutants LiSNFR50.1 (■) and LiSNFR50.3 (▲) have no growth defect compared to wild-type L. infantum (●). Data are means ± SEM from at least three independent experiments. OD600, optical density at 600 nm. (C) Role of AdoMetT1 in SNF resistance. Wild-type L. infantum transfected with empty psp72αHYGα (●) or with LiSNFR50.1 or LiSNFR50.3 transfected with empty psp72αHYGα (■ and ▲, respectively) or with AdoMetT1 cloned into psp72αHYGα (□ and Δ, respectively) were assessed for growth in the presence of increasing concentrations of SNF. Data are means ± SEM for at least three biological replicates. “(H)” in panel C refers to the empty HYG vector.

The drug sinefungin (SNF) was highly effective against Leishmania infantum promastigotes with an *in vitro* 50% effective concentration (EC_50_) of 75 nM. Four independent cultures of wild-type (WT) L. infantum parasites derived from a clone were selected stepwise in liquid medium with increasing concentrations of SNF up to 50 μM. The parasites readily adapted against the drug. The resistant lines were named LiSNFR50.1 to LiSNFR50.4. These mutants were highly resistant to SNF in comparison to the source clonal line (Fig. 1A) and seemed to have no fitness cost for *in vitro* growth as promastigotes (Fig. 1B). When cultured in the absence of SNF for 50 passages, the lines retained resistance against SNF, indicating that it was a stable phenotype (Fig. 1A).

### Whole-genome sequencing and identification of CNVs and SNVs.

**FIG 2:**
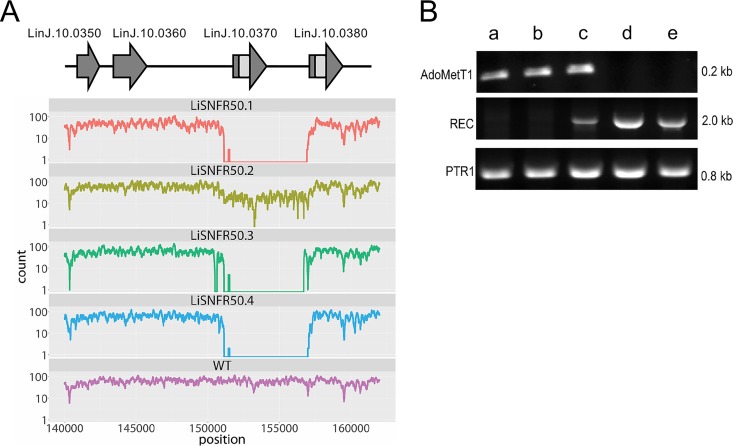
Gene deletion in SNF-resistant mutants mediated by homologous recombination. (A) Sequence read coverage over a 20-kb region on chromosome 10. Depletion of mapped reads over a region of ∼5 kb was observed in the four mutants compared to the wild-type (WT) L. infantum. A subset of folate-biopterin-transporter genes present on chromosome 10 is shown above the map. Regions of perfect identities between *AdoMetT1* (LinJ.10.0370) and LinJ.10.0380 are shown in pale gray. (B) Kinetics of rearrangements at the *AdoMetT1* locus with increasing SNF selection (0 μM [lane a], 0.5 μM [lane b], 1.0 μM [lane c], 2.0 μM [lane d], and 50 μM [lane e]). PCR of *AdoMetT1* or of the novel junction created by the recombination event (REC) between *AdoMetT1* and LinJ.10.0380 were observed using primers shown in Fig. S2C and S2D in the supplemental material. Control PCR was performed with the *ptr1* gene.

Single clones were generated from each of the four highly resistant lines, and their genomic DNAs were sequenced using an Illumina HiSeq2500 platform. Genome coverage for the WT strain and the SNF-resistant mutants varied from 30- to 70-fold (see Fig. S1A in the supplemental material). Read depth coverage over the 36 chromosomes of L. infantum was studied to predict copy number variations (CNVs). Variation in ploidy was observed for specific mutants for chromosomes 10, 21, 31, and 32 but with no common trends (Fig. S1B). One major CNV was detected in all four resistant mutants; this CNV corresponded to a 5-kb deletion in chromosome 10 (Fig. 2A). LiSNFR50.2 differs, however, from the other three mutants. Indeed, while the number of sequence reads is also smaller in this mutant, it is not nil. This could be explained by the heterogeneity of the population with different copy numbers of this region of chromosome 10 that is emerging upon drug selection and passages. The deleted region comprises genes coding for FBT proteins, including the AdoMet transporter (LinJ.10.0370) (Fig. 2A). The deletion of *AdoMetT1* was confirmed by Southern blotting using an *AdoMetT1*-specific probe (Fig. S2A and S2B). Genomic DNAs of *Leishmania* parasites were digested with NheI, and a 2.1-kb band hybridized with the *AdoMetT1* probe in WT cells (Fig. S2B, lane 1) but not in LiSNFR50.1, LiSNFR50.3, or in a resistant cell grown without SNF for several passages (Fig. S2B, lanes 2 to 4). The *AdoMetT1* gene was cloned and transfected in LinSNFR50.1 and LiSNFR50.3 which resulted in a complete reversion of the resistance phenotype (Fig. 1C). Several folate-biopterin-transporter (FBT) genes, including *AdoMetT1*, are located in tandem within a 37-kb region of chromosome 10. These genes possess high level of sequence identities (18, 23). The deletion is characterized by the absence of sequencing reads between nucleotides ∼151 kb and ∼156 kb on chromosome 10 and is flanked by the gene LinJ.10.0380 on its right side (Fig. 2A). We found that recombination occurred within an identical 720-bp region shared between LinJ.10.0370 (*AdoMetT1*) and LinJ.10.0380 (Fig. 2A). Primers were designed to map this rearrangement, and PCR indeed confirmed that the recombination site occurs within this 720-bp region (Fig. S2C and S2D). Sequencing of the rearranged gene further supported the proposed rearrangement (Fig. S3). The *AdoMetT1* rearrangement dynamics was further studied using SNF exposure. Using PCR, we monitored the recombination event between LinJ.10.0370 (*AdoMetT1*) and LinJ.10.0380 and the concomitant disappearance of the *AdoMetT1*-specific amplification. The recombined product started appearing at 2 μM (32× EC_50_) and dominated at higher levels of SNF, with the parallel disappearance of the *AdoMetT1* PCR band (Fig. 2B).

10.1128/mSystems.00416-19.1 FIG S1 Genomic coverage and changes in ploidy in SNF-resistant L. infantum. (A) Average fold genome sequencing coverage for WT and SNF-resistant mutants. For example, on average, every region of the WT strain has been sequenced 70 times, every region of mutant LiSNF50.1 has been sequenced 40 times, and so on. (B) Estimation of ploidy for each chromosome in WT and mutants resistant to SNF. Download FIG S1, TIF file, 1.0 MB. Copyright © 2019 Bhattacharya et al. 2019 Bhattacharya et al. This content is distributed under the terms of the Creative Commons Attribution 4.0 International license.

10.1128/mSystems.00416-19.2 FIG S2 Confirmation of predicted recombination sites for *AdoMet1* deletion. (A) Schematic representation of the *AdoMetT1* locus in L. infantum. NheI restriction sites are shown. The 250-bp DNA probe used for hybridization of Southern blots is shown as a bar below the map. (B) Southern blot of NheI-digested genomic DNAs from WT (lane 1), LiSNFR50.1 (lane 2), LiSNFR50.3 (lane 3), and LiSNFR50.1REV-P50 (lane 4) hybridized with a probe covering 250 bp of the ORF of AdoMetT1 (top panel) or with a probe derived from the gene *ptr1* (bottom panel) used as a loading control. The *ptr1* hybridization was performed on Afe1-digested genomic DNA from the same samples. (C) Homologous recombination between *AdoMetT1* and LinJ.10.0380. Regions of perfect identities are shown in the same shades of color. Primers used for mapping the recombination site and expected sizes are shown (P1 to P5). (D) PCR amplification of DNAs derived from WT (lanes 1) and LiSNFR50.1 (lanes 2) with the set of primers shown in panel C. PCR fragments indicative of gene rearrangement were detected only in LiSNFR50.1. A control PCR with primers against the gene *ptr1* is shown on the right panel. Download FIG S2, TIF file, 0.9 MB. Copyright © 2019 Bhattacharya et al. 2019 Bhattacharya et al. This content is distributed under the terms of the Creative Commons Attribution 4.0 International license.

10.1128/mSystems.00416-19.3 FIG S3 Sequence of the recombinant *AdoMetT1*-LinJ.10.0380 loci. (A) Alignments of *AdoMetT1* and LinJ.10.0380 DNA sequences. (B) Sequence of the recombinant product of *AdoMetT1* and LinJ.10.0380 as predicted by Sanger sequencing of the locus amplified from LiSNFR50.3. The region in black represents identical sequence between the two genes, while regions in blue and red correspond to *AdoMetT1* and LinJ.10.0380, respectively. Download FIG S3, PDF file, 1.7 MB. Copyright © 2019 Bhattacharya et al. 2019 Bhattacharya et al. This content is distributed under the terms of the Creative Commons Attribution 4.0 International license.

The sequence reads were further analyzed for the presence of single-nucleotide variants (SNVs) as previously described (24). Few coding SNVs were detected, and those passing our filtering criteria (see Materials and Methods) were mostly for genes coding for surface proteins, kinesin, or duplicated hypothetical proteins (Table S1), a phenomenon frequently observed in genomics studies on *Leishmania* (19). These were not analyzed further.

10.1128/mSystems.00416-19.9 TABLE S1 List of genes with high-confidence SNVs in at least two independent SNF-resistant mutants. Download Table S1, PDF file, 0.1 MB. Copyright © 2019 Bhattacharya et al. 2019 Bhattacharya et al. This content is distributed under the terms of the Creative Commons Attribution 4.0 International license.

**FIG 3:**
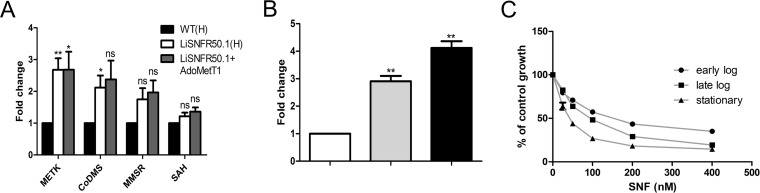
Expression of genes related to AdoMet metabolism and transport. (A) Expression of MetK, cobalamine-dependent methionine synthase (CoDMS), mitochondrial methionine synthase reductase (MMSR), and *S*-adenosylmethionine hydrolase (SAH) were measured in LiSNFR50.1 and compared to WT cells as determined by qRT-PCR. Expression was quantified relative to glyceraldehyde-3-phosphate dehydrogenase (GAPDH). (B) Expression of *AdoMetT1* as measured by qRT-PCR during early (white bar), late logarithmic (gray bar), and stationary (black bar) phases of growth. Expression was quantified relative to GAPDH. (C) Response of *Leishmania* promastigotes to SNF pressure at early log (●), late log (■), and stationary (▲) phases of growth. All data are means plus SEM for three independent replicates. Statistical analyses were performed using paired (A and B) and unpaired (C) two-tailed *t* tests. Statistical significance is indicated as follows: **, *P* < 0.01; *, *P* < 0.05; ns, not significant.

*Leishmania* parasites can both transport and synthesize AdoMet. Since the lack of *AdoMetT1* might affect several important methylation events in the cell, we examined the expression of four SAM-metabolizing enzymes in our SNF-resistant parasites. The genes coding for AdoMet synthetase (MetK) (LinJ.30.3560) and for cobalamin-dependent methionine synthase (CoMS) (LinJ.07.0240) were upregulated in LiSNFR50.1 by 2.5-fold and 2-fold, respectively, but expression of the mitochondrial methionine synthase reductase (MMSR) (LinJ.36.4950) and AdoMet hydrolase (SAH) (LinJ.36.4100) were not significantly altered (Fig. 3A). The episomal expression of *AdoMetT1* did not revert the upregulation of *MetK*. Quantitative reverse transcription-PCR (RT-PCR) revealed alteration of *AdoMetT1* mRNA abundance between the growth phases, attaining maximum at stationary phase (Fig. 3B), and interestingly, in L. infantum promastigotes, SNF responsiveness was determined to be dependent on the growth phase of the inoculum as reflected by the two- to threefold variation in EC_50_ values between early log and stationary phase (Fig. 3C).

### Cos-seq reveals targets for sinefungin along with the diversity of AdoMet function in *Leishmania*.

**FIG 4:**
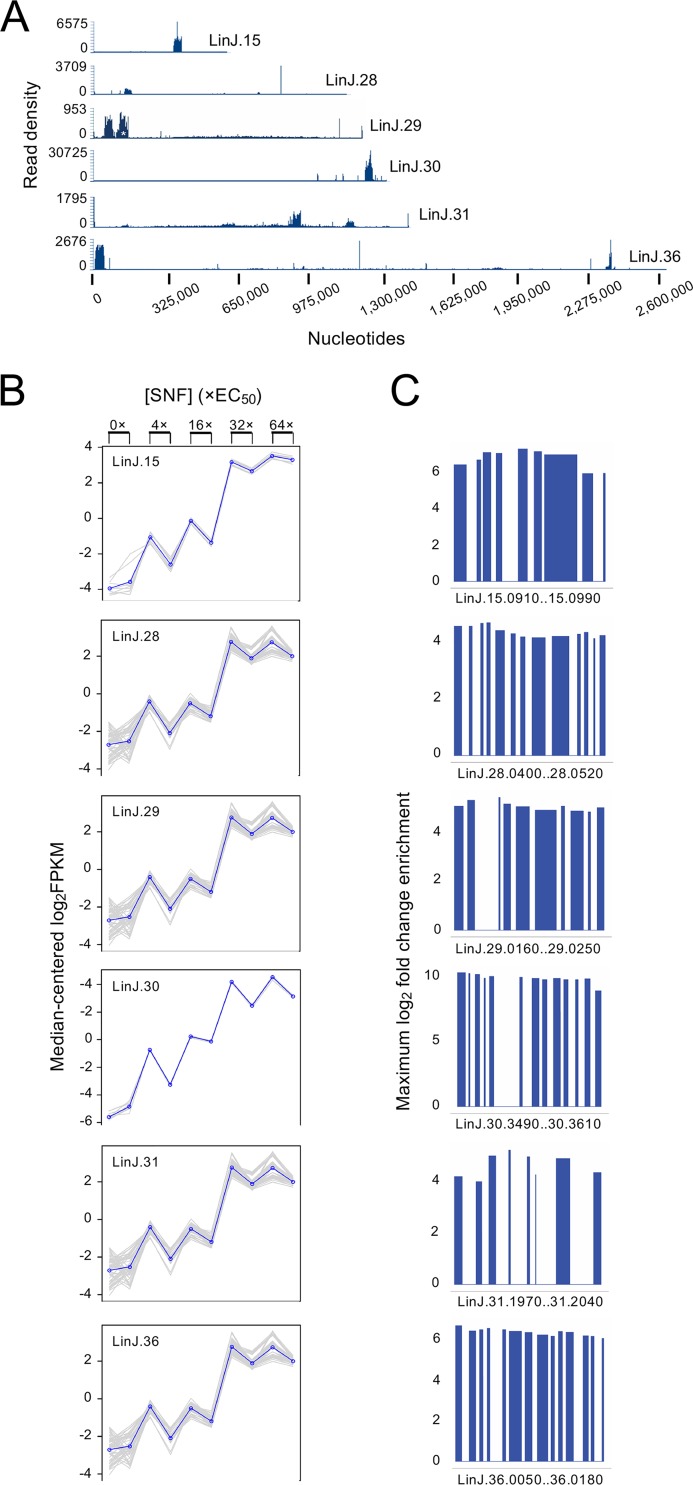
Identification of SNF-responsive loci using a Cos-seq screen with SNF. (A) Visualization of five representative SNF-enriched loci on chromosomes 15, 28, 29, 30, 31, and 36 as delimited by regions of higher read density. A cosmid/region from chromosome 29 that was also enriched in untreated populations through passages is indicated by a white asterisk. (B) Plots of gene clusters sharing similar enrichment profiles. The plots include genes from the five representative SNF-enriched loci recovered by gradual SNF selection. Gray lines represent individual genes, and blue lines denote the average profile per cluster. Gene abundance is expressed on the *y* axis as log_2_ transformed fragments per kilobase of transcript per million mapped reads (FPKM) values centered on the median FPKM. Samples are ordered on the abscissa according to the selection procedure, from nontreated samples (0×) to the final drug increment (64× EC_50_). Gene abundance profiles for the two biological replicates are shown. “Staircase” patterns are due to differences in gene abundance at baseline between the replicates. (C) Maximal fold enrichment for genes from the five enriched cosmids depicted in panel A normalized to the drug-free control. In all cases, maximal enrichment occurred at SNF concentration equivalent to 64× EC_50_.

L. infantum parasites transfected with a cosmid genomic library were selected with SNF at increasing concentrations in biological duplicates. The parasites adapted to each passage and reached stationary phase within 5 days of selection for all doses. Since the parasites showed rapid adaptation to drug selection, cosmids enriched at 4×, 16×, 32×, and 64× EC_50_ selections were sequenced and analyzed. Using a cutoff 16-fold enrichment compared to a similarly cultured but untreated control population, cosmid enrichments of genomic loci derived from chromosomes 15, 28, 29, 30, 31, and 36 were identified, with the highest enrichment being for a locus on chromosome 30 (Fig. 4A to C and Fig. S4). Visualization of cosmid abundance revealed gradual enrichment for each of these, with maximal enrichment occurring at drug selection equivalent to 64× EC_50_ (Fig. 4B). Each enriched cosmid is coding between 8 and 14 genes (Fig. 4C).

### Functional validation of enriched cosmids.

Cosmids isolated from the 64× selection and containing genes derived from chromosomes 15, 28, 30, 31, and 36 were transformed in Escherichia coli and transfected back in L. infantum. Compared to mock-transfected parasites, parasites transfected with the cosmid from chromosome 30 displayed 7.6-fold-higher resistance against SNF, while those with cosmids from chromosomes 31, 36, 15, and 28 showed 4.7-fold, 4.3-fold, 2.9-fold, and 2.8-fold-higher resistance, respectively (Table 1). Individual genes were then overexpressed to pinpoint the specific genes responsible for SNF resistance. Genes with annotated functions were prioritized over hypothetical protein-coding genes. The genes LinJ.30.3560 and LinJ.30.3580 coding for AdoMet synthetase (MetK) conferred sixfold-higher resistance compared to parasite bearing empty vectors, in line with a previous study (12). Two genes located on the cosmid derived from chromosome 36 could be associated with SNF resistance. LinJ.36.0130, coding for the mRNA cap-(guanine-N7)-methyltransferase (CMT1) conferred twofold-higher resistance against SNF (Table 1). The second gene from chromosome 36 was a leucine carboxyl methyltransferase (LCMT) (LinJ.36.0090), which also bestowed twofold-higher resistance against SNF when expressed episomally in WT cells (Table 1). LinJ.31.2000 (TXN3) and LinJ.31.2010 (TXN4), two tryparedoxin-like protein-coding genes, conferred 1.7-fold-higher resistance. Although we could not recover the cosmid derived from chromosome 29, one of its genes encoding a protein phosphatase inhibitor 2 (IPP2) displayed 2.7-fold-higher resistance to SNF when overexpressed in WT parasites (Table 1).

**TABLE 1:** Functional validation of cosmids and genes for their resistance against SNF^*a*^

Chromosome	Cosmid start position	Cosmid end position	Fold resistance for cosmid^*b*^	Resistance gene on cosmid	Gene product	Fold resistance for gene^*b*^
15	373198	408034	2.91 ± 0.38*** (*n* = 3)	LinJ.15.0980	PP2AR	1.53 ± 0.13** (*n* = 5)
30	1266355	1301597	7.60 ± 1.98*** (*n* = 6)	LinJ.30.3560	METK	5.99 ± 1.63*** (*n* = 6)
31	929321	964656	4.72 ± 0.54*** (*n* = 3)	LinJ.31.2000	Trxn-like	1.70 ± 0.11*** (*n* = 3)
				LinJ.31.2010	Trxn-like	1.75 ± 0.06*** (*n* = 3)
29	ND	ND	ND	LinJ.29.0180	IPP2	2.70 ± 0.55*
28	140575	172918	2.79 ± 0.45***	ND	ND	ND
36	47576	11107	4.30 ± 0.47*** (*n* = 3)	LinJ.36.0090	LCMT	2.03 ± 0.17*** (*n* = 3)
				LinJ.36.0130	CMT1	2.19 ± 0.40*** (*n* = 5)

a Cosmids and genes enriched by Cos-seq were functionally tested in WT cells by episomal expression and growth curve assays against SNF. ND, not determined.

b The fold resistance to SNF is expressed as the ratio of the EC_50_s for parasites transfected with the target gene or cosmid to the EC_50_s for mock-transfected parasites. Values are means ± standard deviations (SD) from at least three independent experiments. The *P* values were calculated by two-tailed unpaired *t* test and are indicated by asterisks as follows: ***, *P* < 0.001; **, *P* < 0.01.

**FIG 5:**
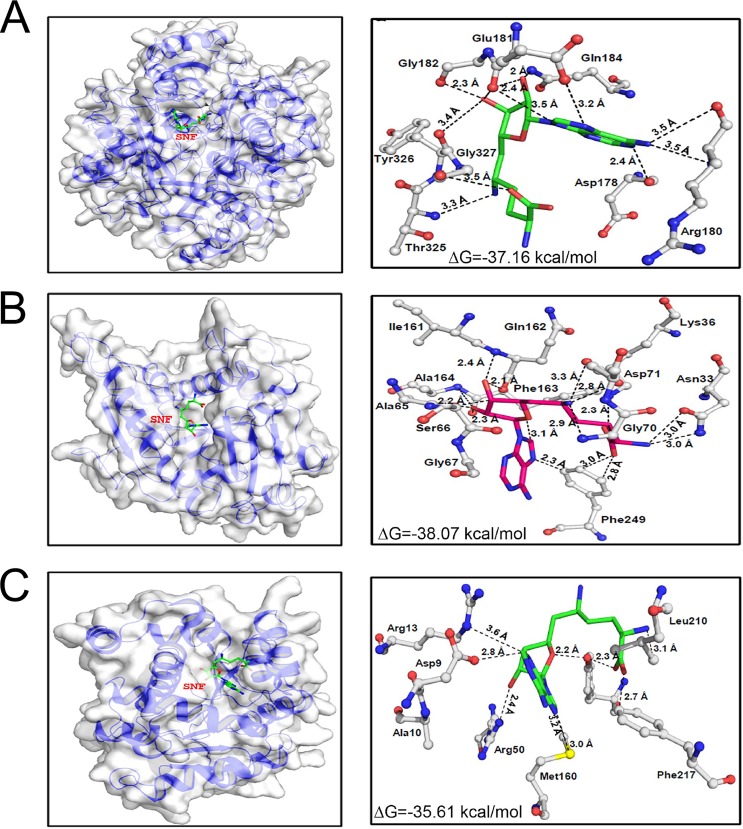
Model structures of targets identified by Cos-seq docked with SNF. (A to C) SNF binding pockets and specific binding residues for MetK (A), CMT1 (B), and LCMT (C). The binding site residues were identified from the LigPlot+ representations illustrating hydrophobic and hydrogen-bond interactions with SNF. The amino acid residues are represented as a ball and stick. Elements are shown in color as follows: white (carbon), blue (nitrogen), and red (oxygen). SNF atoms are represented in green (carbon), blue (nitrogen), and red (oxygen).

At least three gene products isolated from the Cos-seq screen, namely, MetK, CMT1, and LCMT, contain AdoMet binding sites as determined by molecular docking modeling of SNF and AdoMet with these targets. Docking of MetK revealed a binding affinity for SNF of −37.16 kcal/mol. It formed 11 hydrogen bonds (H-bonds) and hydrophobic interactions (Fig. 5). The binding site is similar to the docking site of AdoMet with at least two conserved H-bonds (Q184 and Y326) (Fig. S5). CMT1 showed similar binding affinity for SNF (−38.05 kcal/mol) with at least 13 potential H-bond and hydrophobic interactions. A binding affinity of −35.01 kcal/mol was showed by LCMT with SNF binding with at least eight H-bonds and hydrophobic interactions (Fig. 5). Binding of AdoMet by CMT1 and LCMT shares several conserved interactions as observed for SNF (Fig. S5).

10.1128/mSystems.00416-19.4 FIG S4 Genomewide distribution of SNF-enriched loci derived from the Cos-seq screen. Genomic regions significantly enriched by SNF as revealed by Cos-seq are shown for each of the 36 *Leishmania* chromosomes. Gray bars represent the gene positions on each chromosome. Colored bars represent genes enriched at 64× SNF EC_50_ classified by log_2_ transformed enrichment levels according to the color code shown on the right. Download FIG S4, TIF file, 0.8 MB. Copyright © 2019 Bhattacharya et al. 2019 Bhattacharya et al. This content is distributed under the terms of the Creative Commons Attribution 4.0 International license.

10.1128/mSystems.00416-19.5 FIG S5 The molecular model structure of targets identified by Cos-seq docked with AdoMet. AdoMet binding pockets and specific binding residues for METK (A), CMT1 (B), and LCMT (C) are shown. The binding site residues were identified from the *LigPlot*+ representations illustrating hydrophobic and hydrogen-bond interactions with SNF. The amino acid residues are represented as a ball and stick and in the following colors: white (carbon), blue (nitrogen), and red (oxygen). AdoMet-atoms are shown in color as follows: magenta (carbon), blue (nitrogen), red (oxygen), and yellow (sulfur). Download FIG S5, TIF file, 2.4 MB. Copyright © 2019 Bhattacharya et al. 2019 Bhattacharya et al. This content is distributed under the terms of the Creative Commons Attribution 4.0 International license.

### Leucine carboxyl methyltransferase and sinefungin.

**FIG 6:**
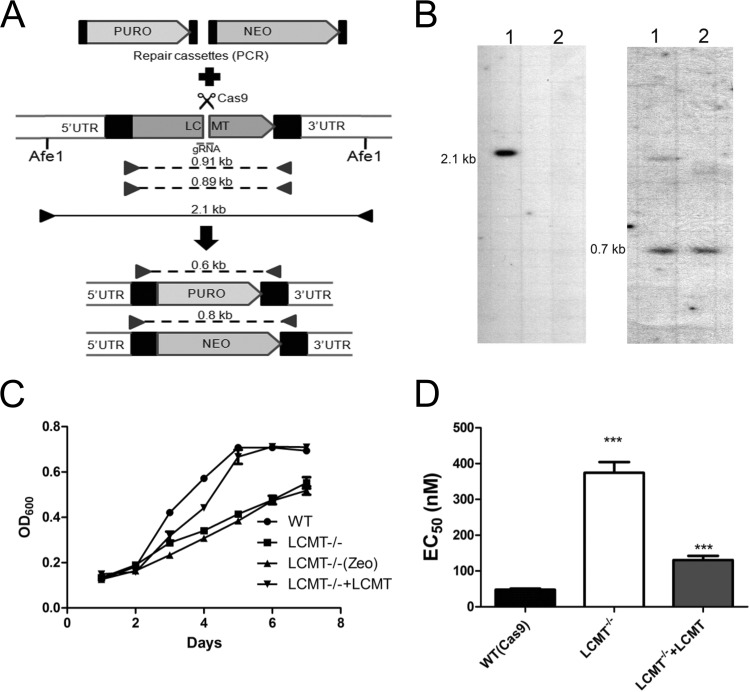
Gene knockout of the *Leishmania* leucine carboxyl methyltransferase LCMT. (A) CRISPR-Cas9-assisted gene knockout of LCMT in L. infantum cells expressing Cas9 (*Li*Cas9). Puromycin (PURO) and neomycin (NEO) repair cassettes were generated by PCR using primers with overhangs corresponding to the first 30 nucleotides (nt) of the LCMT 5′ and 3′ untranslated regions (UTRs) for integration by homologous recombination at the cut site. The repair templates were transfected in *Li*Cas9 along with a gRNA-crRNA hybrid before the selection of transfectants with puromycin or G418. The puromycin- or G418-selected populations were then plated, and five individual clones were isolated for each transfection. (B) Confirmation of LCMT knockout was obtained by Southern blotting analysis where genomic DNAs from WT (*Li*Cas9) (lanes 1) and LCMT^−/−^ cells (lanes 2) were digested with Afe1 and hybridized to LCMT-specific (left) or PTR1-specific (right) DNA probes. (C) The growth of WT L. infantum, LCMT^−/−^, and LCMT^−/−^ add-back was monitored in SDM medium for 7 days by OD_600_ measurements. Data are means ± SEM from at least three independent experiments. (D) L. infantum LCMT^−/−^ parasites are resistant to SNF as determined by EC_50_ measurements for WT (*Li*Cas9) and LCMT^−/−^ or LCMT^−/−^ add-back cells. Data are means plus SEM for at least three biological replicates. Statistical analyses were performed using unpaired two-tailed *t* tests. ***, *P* < 0.001.

The role of leucine carboxyl methyltransferase (LCMT) is to methylate the carboxyl group of C-terminal leucine residues of proteins. More than 10% of total proteins in L. infantum contain leucine at their C termini (Fig. S6A). Multiple alignment with bacterial and higher eukaryotic orthologs of LCMT demonstrated that LCMT from L. infantum and Trypanosoma brucei shares significant amount of conservation (Fig. S6B), although the kinetoplastid LCMTs cluster distantly from eukaryotic LCMTs (Fig. S6C). The LCMT gene was inactivated by integrating neomycin and puromycin resistance cassettes using a CRISPR-Cas9-based approach (21) in L. infantum (Fig. 6A). The knockout was confirmed by Southern blotting (Fig. 6B) and by PCR using open reading frame (ORF)-specific and untranslated region (UTR)-specific primers (Fig. S7A). Although LCMT^−/−^ cells grew well in SDM medium, the growth in log phase was found to be impaired compared to WT (Cas9) cells (Fig. 6C). The phenotype was reverted by episomal expression of LCMT (Fig. 6C). LCMT^−/−^ cells were unexpectedly four- to fivefold more resistant to SNF (Fig. 6D). The resistance was abolished when LCMT was expressed episomally in LCMT^−/−^ cells (Fig. 6D). This is in contrast to the Cos-seq screen, where LCMT episomal overexpression led to resistance. This apparent dichotomy may be explained by the role of LCMT during growth phase. Indeed, while LCMT^−/−^ cells are resistant to SNF both in log and stationary phase, cells expressing LCMT as part of an episome displayed maximum resistance in early log phase, and the resistance is gradually lost in late-log and stationary-phase promastigotes (Fig. S7B).

10.1128/mSystems.00416-19.6 FIG S6 Amino acid composition at the C termini of L. infantum proteins and leucine carboxyl methyltransferase (LCMT) evolution. (A) The percentages of proteins harboring specific C-terminal amino acid residues were scored for the 8,239 proteins annotated in the genome of L. infantum. (B) Multiple sequence alignment of amino acid sequences from LCMTs from various organisms. The LCMTs were from Acinetobacter baumannii (Ac) (NCBI:protein accession no. ATP87955.1), Saccharomyces cerevisiae (Sc) (pdb|1RJD|C Chain C), Leishmania infantum (Li) (LinJ.36.0090), Trypanosoma brucei (Tb) (Tb927.10.4460), Homo sapiens (Hs) (NCBI:protein accession no. AAF18267.1), Caenorhabditis elegans (Ce) (sp|P46554), Acanthamoeba castellanii (Ac) (ELR11879.1), Plasmodium falciparum (Pf) (PKC49946.1), and Arabidopsis thaliana (At) (AEE27384.1). Multiple sequence alignment was performed by CLUSTALΩ, and the alignment was visualized using BioEdit. Black shades signify identity across sequences. (C) Phylogenetic analysis of LCMT. A Newik phylogenetic tree was constructed from the CLUSTALΩ alignment with *MEGA6* by the neighbor-joining method with 10,000 bootstraps, computed by JTT analysis. Distances and bootstrap values are depicted at each branch node depicting the evolutionary proximity of the orthologues. Download FIG S6, TIF file, 1.8 MB. Copyright © 2019 Bhattacharya et al. 2019 Bhattacharya et al. This content is distributed under the terms of the Creative Commons Attribution 4.0 International license.

10.1128/mSystems.00416-19.7 FIG S7 Knockout of LCMT using CRISPR-Cas9. (A) CRISPR-Cas9-assisted gene knockout of *LCMT* was attempted in L. infantum cells expressing Cas9 (*Li*Cas9). The *LCMT* knockout was validated by PCR from genomic DNAs derived from WT L. infantum (lane 1) or from five putative LCMT knockout clones (lanes 2 to 6) using primers designed from LCMT ORF and UTRs as depicted in Fig. 6A. (B) The susceptibility to SNF of WT L. infantum, LCMT^−/−^, and LCMT^−/−^ expressing LCMT-HA was determined at various phases of growth. Download FIG S7, TIF file, 0.1 MB. Copyright © 2019 Bhattacharya et al. 2019 Bhattacharya et al. This content is distributed under the terms of the Creative Commons Attribution 4.0 International license.

**FIG 7:**
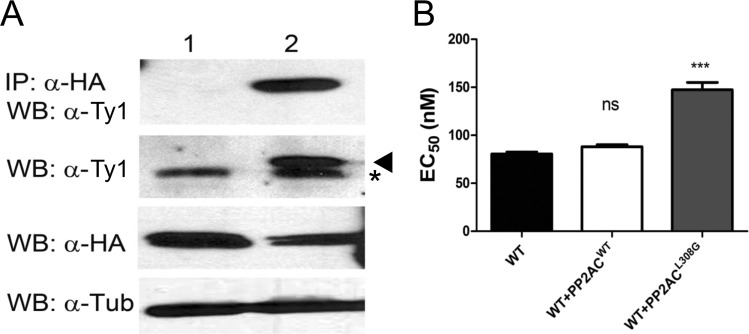
Interactions between LCMT and the protein phosphatase PP2AC. (A) HA-tagged LCMT was expressed either alone (lane 1) or coexpressed along with 2×-Ty1-tagged PP2AC (lane 2) in LCMT^−/−^ cells. Immunoprecipitation (IP) was performed using mouse anti-HA (α-HA) coupled magnetic beads. Immunoprecipitates were tested for the presence of PP2AC by immunoblotting (Western blotting [WB]) using rabbit anti-Ty1 antibody (α-Ty1) (top panel). The level of expression of 2×-Ty1-PP2AC (second panel) (marked with an arrowhead) and LCMT-HA (third panel) was tested by immunoblotting using anti-Ty1 and anti-HA antibodies, respectively. Our anti-Ty1 antibody reacts with an unknown 30-kDa protein, which is marked with an asterisk. α-Tubulin (α-Tub) was detected as loading control (bottom panel). (B) Impact of PP2AC on SNF responsiveness was studied by expressing PP2AC^WT^ and PP2AC^L308G^ in L. infantum WT cells. EC_50_ values were determined by dose-response curves against SNF. Data are means plus SEM for at least three biological replicates. Statistical analyses were performed using unpaired two-tailed *t* tests. ***, *P* < 0.001; ns, not significant.

In eukaryotes, LCMT is known to methylate and regulate PP2A catalytic subunit (PP2AC) and related protein phosphatases (25). Intriguingly, cosmids derived from chromosomes 15 and 29 isolated from the Cos-seq screen carry genes that encode regulators of phosphatase. In order to determine whether PP2AC and LCMT do interact, a C-terminal hemagglutinin (HA)-tagged version of LCMT was cotransfected with a N-terminal Ty1-tagged PP2AC in LCMT^−/−^ parasites. Immunoprecipitation with anti-HA antibody followed by Western blotting with anti-Ty1 antibody confirmed that LCMT interacts with PP2AC (Fig. 7A). To further investigate the LCMT-PP2AC interactions, we carried out high-ambiguity-driven protein-protein docking (26) with quality models of the *Leishmania* LCMT and PP2AC. The model for the *Leishmania* predicted complex structure exhibited similarity in orientation and organization with crystal structure of the human LCMT1 and PP2AC complex with the C-terminal leucine of PP2AC buried inside LCMT for both structures (Fig. S8). Analysis of the interaction interface and stabilization energy revealed that both the proteins have considerable continuous patches of interaction with stabilization energy of −661.6545 kJ/mol, indicating strong interaction between the two proteins (Fig. S8). To examine whether methylation of the C-terminal leucine of PP2AC (L308) is indeed linked to the SNF response, we independently transfected WT L. infantum parasites with episomes coding for WT PP2AC or for a PP2AC^L308G^ variant in which the C-terminal leucine was mutated to a glycine residue. The PP2AC^L308G^-expressing parasites elicited 1.75-fold-higher resistance compared to cells expressing the WT version of PP2AC (Fig. 7B).

10.1128/mSystems.00416-19.8 FIG S8 Interactions of LCMT with PP2AC as determined by high-ambiguity-driven protein-protein docking (HADDOCK). (A) The predicted structure of LiLCMT is shown in blue and the predicted structure of LiPP2AC is shown in orange with LEU-308 labeled. (B) The human LCMT1-PP2AC structure is presented with HsLCMT1 in green and HsPP2AC in red. Side view (C) and top view (D) of the predicted interface of LiLCMT (blue) and PP2AC (orange) interactions are shown in mesh. (E) The patch areas of interactions are listed. Download FIG S8, TIF file, 2.6 MB. Copyright © 2019 Bhattacharya et al. 2019 Bhattacharya et al. This content is distributed under the terms of the Creative Commons Attribution 4.0 International license.

## DISCUSSION

SNF is a nucleoside antibiotic structurally related to AdoMet that competitively inhibits AdoMet-synthesizing and -dependent enzymes (27). The antileishmanial activity of SNF is well established (15), but the drug was not further developed due to nephrotoxicity and toxicity to bone marrow cells (28, 29). Nonetheless, work is still ongoing in developing SNF analogues with higher therapeutic indexes (29–32). This is certainly justified in light of the recent discovery that lead antitrypanosomal boron-containing compounds perturb AdoMet metabolism and seem to act similarly to SNF (33).

Whole-genome sequencing of independent resistant mutants could detect a deletion of AdoMetT1, the AdoMet transporter. This is reasonable, as SNF was previously shown to use the AdoMet transporter in *Leishmania* (16, 17). This paralleled observations made in SNF-resistant yeast or Toxoplasma gondii where mutations in their AdoMet transporters were found to be the main driver of resistance (34, 35). However, in those cases, the AdoMet transporters are part of the amino acid permease superfamily, and resistance was mediated by point mutations rather than gene deletion as in our L. infantum SNF-resistant mutants. Our next-generation sequencing (NGS) work allowed us to precisely define the molecular mechanism of gene deletion that was mediated by homologous recombination between conserved regions of FBT genes, a frequent mechanism of gene rearrangement in *Leishmania* (36). The deletion took place in the same region between *AdoMetT1* and LinJ.10.0380 in all four mutants, despite the presence of other homologous repeats within the FBT paralogues of chromosome 10. One possible reason could be the fitness cost that the loss of folate transport would have brought from the deletion of the nearby FT1 or FT5 (23, 37). The *AdoMetT1* gene is preferentially expressed in stationary phase that is correlated with SNF susceptibility (Fig. 3). Possibly *Leishmania* uses primarily the AdoMet biosynthetic route during logarithmic phase, but because of metabolic reprogramming during stationary phase, the parasite may rely to a greater extent on transport of AdoMet to meet its AdoMet requirements. No phenotypic SNVs could be associated with SNF resistance in our NGS effort.

Analysis of the resistant mutants led to an understanding of the main strategy to resist SNF, but we had to rely on a functional genomic screen to isolate genes that could help in our understanding of the physiological role of AdoMet in *Leishmania*. A Cos-seq screen (20) highlighted the enrichment of at least six cosmids by SNF selection and identified MetK as a target for SNF in *Leishmania*. This gene produced six- to sevenfold-higher resistance to SNF. This is lower than the last drug concentration (64×) used during the Cos-seq selection, but our experience indicates that we can seldom reach the level of resistance used for selection while transfecting individual genes. It is salient to point out, however, that even if cells are selected at 64× EC_50_, they may not be resistant to that level of drug. Indeed, during continuous drug selection, a population may arise where there is transient physiological adaptation or tolerance that may facilitate growth in the presence of the drug. Of the other cosmids/genes that were revealed, one was coding for the mRNA cap-(guanine-N7)-methyltransferase CMT1. SNF is known to inhibit several viral N7 cap methyltransferases (38) and fungal enzymes (39), and it was suggested to be the target of SNF antifungal activity (40). Cotransfection of the yeast MetK (SAM1) and N7 cap methyltransferase (ABD1) was shown to produce resistance to SNF in yeast (34). Work on CMT1 has been carried out in the related parasite Trypanosoma brucei (41), and this gene appears to be nonessential (42). Here, we suggest, in line with viral work, that along with MetK the *Leishmania* CMT1 may be a secondary target for SNF. Modeling studies (Fig. 5) support this suggestion.

Our Cos-seq screen also led to the characterization of the leucine carboxyl methyltransferase LCMT. Molecular docking studies identified crucial residues for the interaction between AdoMet or SNF with LCMT as well (Fig. 5; see also Fig. S5 in the supplemental material). In mammals, LCMT-1 methylates the C-terminal leucine of the C-subunits of protein phosphatases of the PP2A subfamily. Its methylation facilitates the formation of PP2A heterodimers that are involved in a plethora of physiological processes related to cell growth and proliferation (43). PP2AC-LCMT interactions were verified in *Leishmania* by immunoprecipitation of the two coexpressing tagged versions of the proteins. In contrast to *Leishmania*, LCMT is essential in mice (44). Overexpression of LCMT produces SNF resistance (Table 1), but its inactivation produced even more resistance (Fig. 6C). We found that overexpression of LCMT produces significant resistance only in early log phase of growth, while the LCMT^−/−^ cells are resistant to SNF at every growth phase of the parasite. Thus, one possible explanation is that the *Leishmania* protein has several targets and their state of methylation (possibly linked to growth phase) is implicated in SNF resistance. For example, replacement of the terminal leucine in PP2AC contributed to SNF resistance (Fig. 7B). Interestingly, about 10% of the proteins in L. infantum possess leucine residues at their C termini. The *Leishmania* LCMT is 28% identical to the mammalian enzyme and phylogenetically distinct among LCMT orthologues, and it remains to be seen whether it also has PP2A as a substrate. Protein-protein docking would suggest that this is quite possible (Fig. S8). It is intriguing that the Cos-seq screen led to a PP2A regulatory subunit (LinJ.15.0980) which was found to elicit (low) resistance against SNF (Table 1). The LCMT^−/−^ mutant is resistant to SNF (Fig. 6C), and this is consistent with our observation that WT cells with episomal expression of a PP2A^L308G^ version were also slightly resistant to SNF. The protein LinJ.29.0180, also involved in the response to SNF, has a Pfam motif for protein phosphatase inhibitor 2, and possibly the modulation in the activity of a number of phosphatases influences AdoMet metabolism and thus, the SNF response.

Two genes encoding tryparedoxin (TXN3 and TXN4) were also shown to contribute to SNF resistance (Table 1). Overexpression of tryparedoxin possibly helped the parasite to circumvent the redox imbalance imposed by SNF.

The use of independent genomic approaches for studying the mode of action of SNF in *Leishmania* allowed the characterization of novel features related to AdoMet function in *Leishmania*. SNF has interesting activity against *Leishmania*, and reducing its toxicity may bring it further along the development pipeline. Potential targets have now been found which could further help in the development of SNF-like compounds. Ideally, these analogues would maintain specific activity against multiple *Leishmania* targets while being more lipophilic, hence escaping the need to enter the cells through AdoMetT1, a locus frequently deleted when cells are in contact with SNF.

## MATERIALS AND METHODS

### Parasite culture.

L. infantum MHOM/MA/67/ITMAP-263 parasites and the L. infantum MHOM/MA/67/ITMAP-263 population harboring a cosmid library (20) were maintained as promastigotes at 25°C in SDM-79 or M199 as described earlier (20). Cell growth and 50% effective concentration (EC_50_) were monitored by measuring the absorbance at 600 nm as described before (20).

### Whole-genome sequencing and analysis.

Paired-end sequencing libraries were prepared from L. infantum genomic DNA with the Nextera DNA sample prep kit and sequenced on an Illumina HiSeq platform with 101-nucleotide paired-end reads. An average genome coverage of more than 50-fold was achieved (see Fig. S1A in the supplemental material). Sequence reads were aligned to the L. infantum JPCM5 genome using bwa-mem (45). Read duplicates were marked using Picard, and GATK was applied for discovering single-nucleotide variants (SNVs) and small insertions or deletions (indels) (46). SNVs and indels from the vcf files were filtered using the following hard filtering criteria: mappingQual (MQ) of ≥40, FisherStrand (FS) of ≤60, QualByDepth (QD) of ≥5, MappingQualityRankSumTest (MQRankSum) of ≥−2.5, and ReadPosRankSumTest (ReadPosRankSum) of ≥−4. Single-nucleotide variants (SNVs) revealed by next-generation sequencing (NGS) were confirmed by PCR amplification and conventional DNA sequencing. Copy number variations (CNVs) were derived from read depth coverage as described earlier (47).

### Cosmid extraction, purification, and paired-end sequencing library preparation.

Cosmid extraction was conducted as previously described (20). Purified total DNA was treated with RiboShredder RNase blend (Epicentre) to remove potential RNA contaminations. Genomic DNA was removed with plasmid-safe ATP-dependent DNase (Epicentre) following the manufacturer’s instructions. In addition, kinetoplastid DNA was removed by electrophoresis of DNase-treated cosmid extracts on 1% low-melting-point agarose (Invitrogen) followed by excision and purification of the bands corresponding to high-molecular-weight cosmid DNA (∼50 kb). Purified cosmid DNA was quantified with the QuantiFluor dsDNA system staining kit (Promega). Fifty nanograms of purified cosmid DNA was used for paired-end library preparation using Nextera DNA sample preparation kit (Illumina). Sequencing libraries were quantified with the QuantiFluor dsDNA system and sequenced using an Illumina HiSeq system at a final concentration of 8 pM.

### Cosmid enrichment analysis.

Sequencing reads from each sample were independently aligned to the L. infantum JPCM5 reference genome (version 8.0) obtained from TritrypDB (http://tritrypdb.org/tritrypdb/) using the bwa-mem software (48). BAM files were converted to BED files by using BEDTools (49), and the read depth and genome coverage were visualized using the SignalMap software (Roche NimbleGen). The detection of enriched genes relied on the Trinity software version 2.1.1 (50), which includes all third-party tools required for the analysis. Gene abundance within samples was quantified using the kallisto software (51). Clusters of genes significantly enriched by drug selection were retrieved with edgeR (52) using the default parameters (false-discovery rate of ≤0.001). Gene clusters were then plotted according to the median-centered log_2_-transformed fragment per kilobase per million mapped reads (FPKM) values using R scripts included in the Trinity package. Only genes with a log_2_ fold change of ≥4 were retained. The cosmid fold enrichment was computed by extracting the mean FPKM ratio for the genes on enriched cosmids in the drug-selected samples normalized to the mean FPKM ratio for these genes in the control sample passaged in the absence of drug.

### DNA constructs, cosmid isolation, and transfection.

The genes of L. infantum were amplified from genomic DNA using compatible primer pairs and cloned in the *Leishmania* expression vectors pSP72αZeoα, pSP72αPuroα, or pSP72αHYGα unless mentioned otherwise. A total of 20 μg of plasmid DNA for episomal expression was transfected into *Leishmania* promastigotes by electroporation.

The enriched cosmids used for paired-end sequencing library preparation were transformed in Escherichia coli DH5α and were either recovered by random picking of transformed colonies or by colony hybridization as described earlier (20). Candidate cosmids were transfected in wild-type (WT) L. infantum parasites.

Knockout cell lines were generated in WT L. infantum expressing Cas9 (*Li*Cas9) (21). Puromycin and neomycin resistance genes were amplified with primers containing 30- to 40-bp sequences upstream and downstream of the target gene. A CRISPR RNA (crRNA) targeting the open reading frame (ORF) of LCMT (LinJ.36.0090) was designed targeting the following sequence: gRNALCMT, GCGACCTGTATGACGCCAGG. The guide RNA (gRNA) was generated by hybridizing 5 μl of 0.1 nmol/μl crRNA with 5 μl of equimolar *trans*-activating crRNA (tracrRNA) (IDT) as described earlier (21). Eight micrograms of each repair template and 5 μl of each crRNA-tracrRNA hybrid were transfected simultaneously using Amaxa Nucleofector transfection kit (Lonza). The selection was done with puromycin at 100 μg/ml or with neomycin at 400 μg/ml. Allelic substitutions were confirmed by PCR amplification of target genes followed by standard sequencing.

### Immunoprecipitation and Western blot analysis.

Immunoprecipitation was done using Pierce HA-tag magnetic IP/co-IP kit according to the manufacturer’s protocol. Lysis of pellets derived from mid-log-phase cells was performed using lysis buffer supplemented with Halt protease inhibitor cocktail and Halt phosphatase inhibitor cocktail (Thermo Scientific) with 20 to 30 strokes of a Dounce homogenizer with the cells on ice. Clear supernatants obtained after centrifugation (10,000 × *g*; 30 min) were incubated with antihemagglutinin (anti-HA) magnetic beads at 4°C for 4 h on a gentle rotator. The beads were separated and washed, and SDS-PAGE was performed on 10% or 12% acrylamide gels by standard procedures. Protein expression Immobilon western chemiluminescence kit (Millipore, Billerica, MA, USA) was used to detect proteins. Antibodies and dilutions used are as follows: mouse anti-HA IgG (Santa Cruz), (1:5,000), mouse antitubulin IgG (Millipore) (1:5,000), horseradish peroxidase (HRP)-conjugated anti-mouse IgG (Cell Signaling) (1:10,000), rabbit anti-Ty-1 IgG (Genscript) (1:500), and HRP-conjugated goat anti-rabbit IgG (GE Life Sciences) (1:5,000).

### Homology model and molecular docking.

The homology model structures of METK (LinJ.30.3560), CMT1 (LinJ.36.0130), andLCMT (LinJ.36.0090) are built using the protein template structures from the Protein Data Bank (PDB) entries or accession nos. 4ODJ, 4FYU, 3IEI, and 5E8J. The model structures were built using a fully automated protein structure homology modeling server SWISS-MODEL (http://swissmodel.expasy.org/) (53). The model quality was estimated based on the QMEAN scoring functions of 0.80, 0.55, 0.68, and 0.56 which are within the acceptable range (54). PyMOL v1.3 was used to visualize the structural models (55). *In silico* docking of structural models of METK, CMT1, and LCMT with SNF and AdoMet was conducted using the PATCHDOCK server (56) and FireDock, an efficient method for the refinement and rescoring of rigid-body docking solutions (57). The binding site residues are identified from the LigPlot+ (58) for representation of hydrophobic and hydrogen-bond interactions.

### Protein-protein docking.

Homology models for LiLCMT and PP2A individually were built using SWISS-MODEL (59) with complex structure of human LCMT-1 and PP2ACα as the template with QMEAN scores of −1.84 and −1.59, which are within the allowable limit. Each of the structures was validated by Procheck (60) with one and two residues in the disallowed region of the Ramachandran plot. Docking was performed with the two models using HADDOCK (26) after defining the restraints using CPORT (61). The best-docked cluster with a root mean square deviation (RMSD) of 0.4 ± 0.2 was further refined with Galaxy Refine Complex (62). The interface and stabilization energies of the complex were analyzed by Proface (63) and PIMA (64), respectively.

### Statistical analysis.

For statistical analysis, two-tailed unpaired *t* test with GraphPad Prism 5.01 software was performed unless mentioned otherwise.

### Availability of data and materials.

The data set supporting the conclusions of this article is available in the Sequencing Read Archive (https://www.ncbi.nlm.nih.gov/sra) repository under BioProject accession no. PRJNA552229 and sample accessions SAMN12184655 to SAMN12184659.
