# Supplementary figures and images for: Genomewide Analysis of Mode of Action of the S-Adenosylmethionine Analogue Sinefungin in Leishmania infantum
Source: mSystems. 2019 Oct 15;4(5):e00416-19. doi: 10.1128/mSystems.00416-19 (PMC6794121; doi:10.1128/mSystems.00416-19)

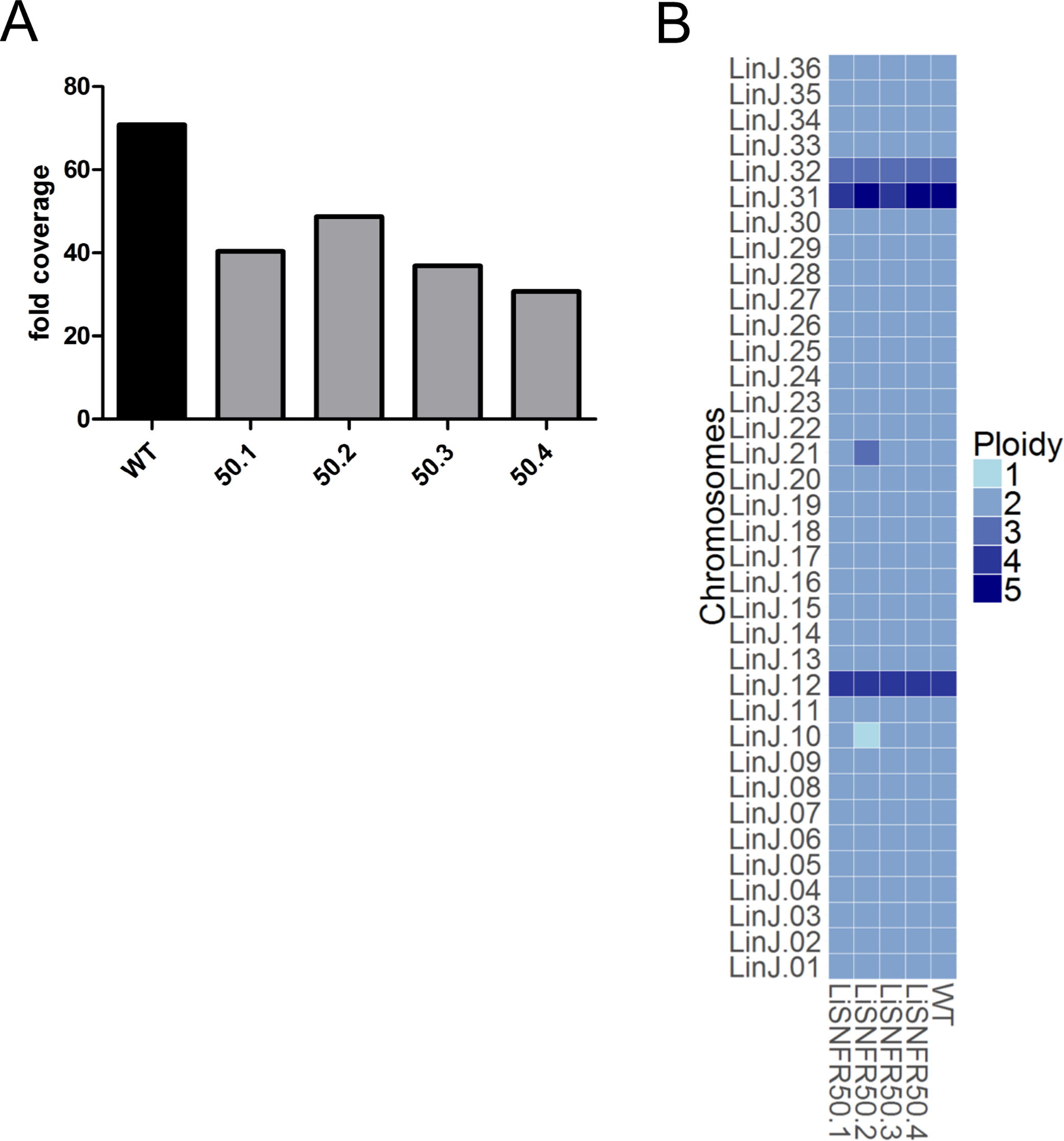

Supplement: FIG S1 [file mSystems.00416-19-sf001.tif]

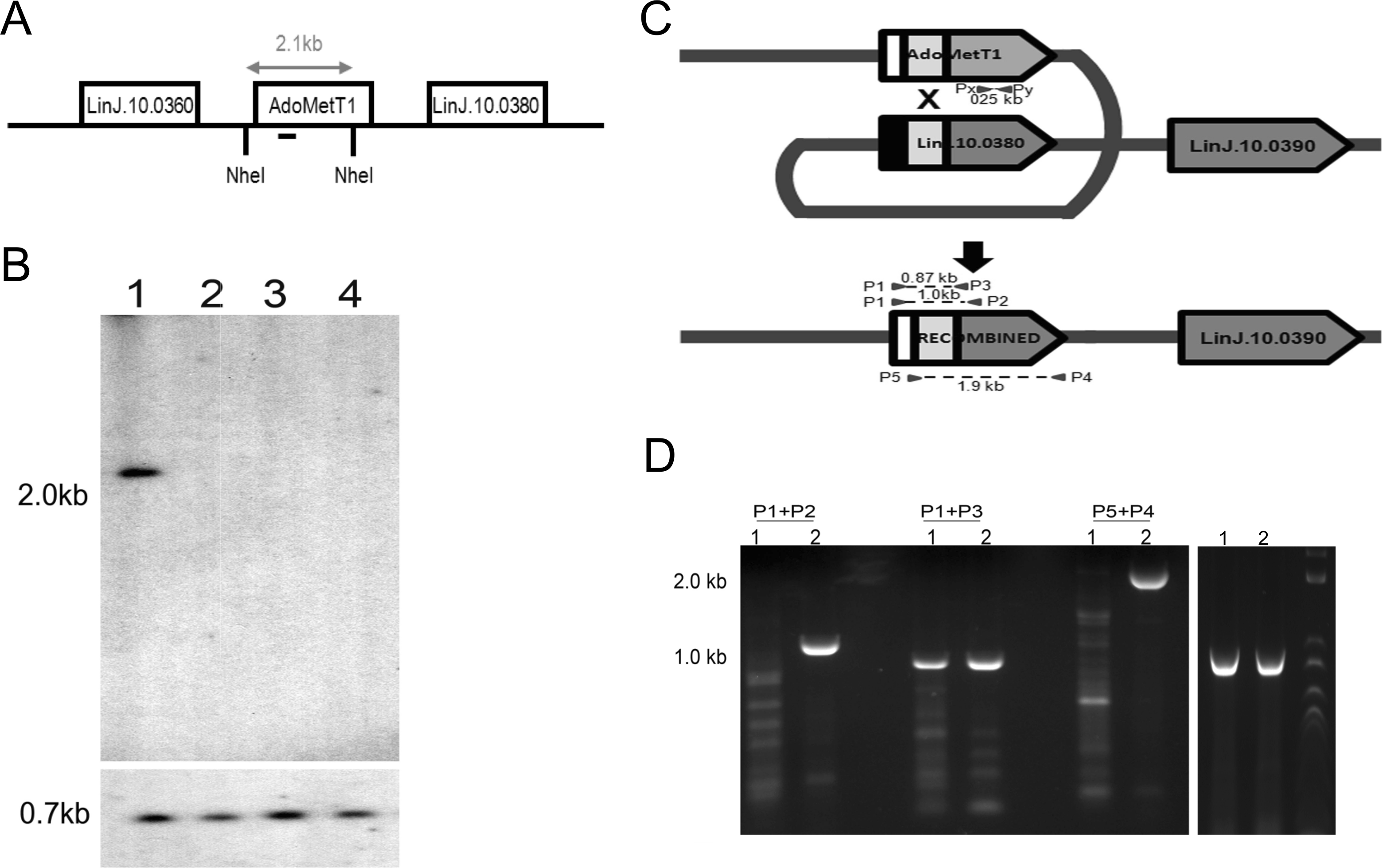

Supplement: FIG S2 [file mSystems.00416-19-sf002.tif]

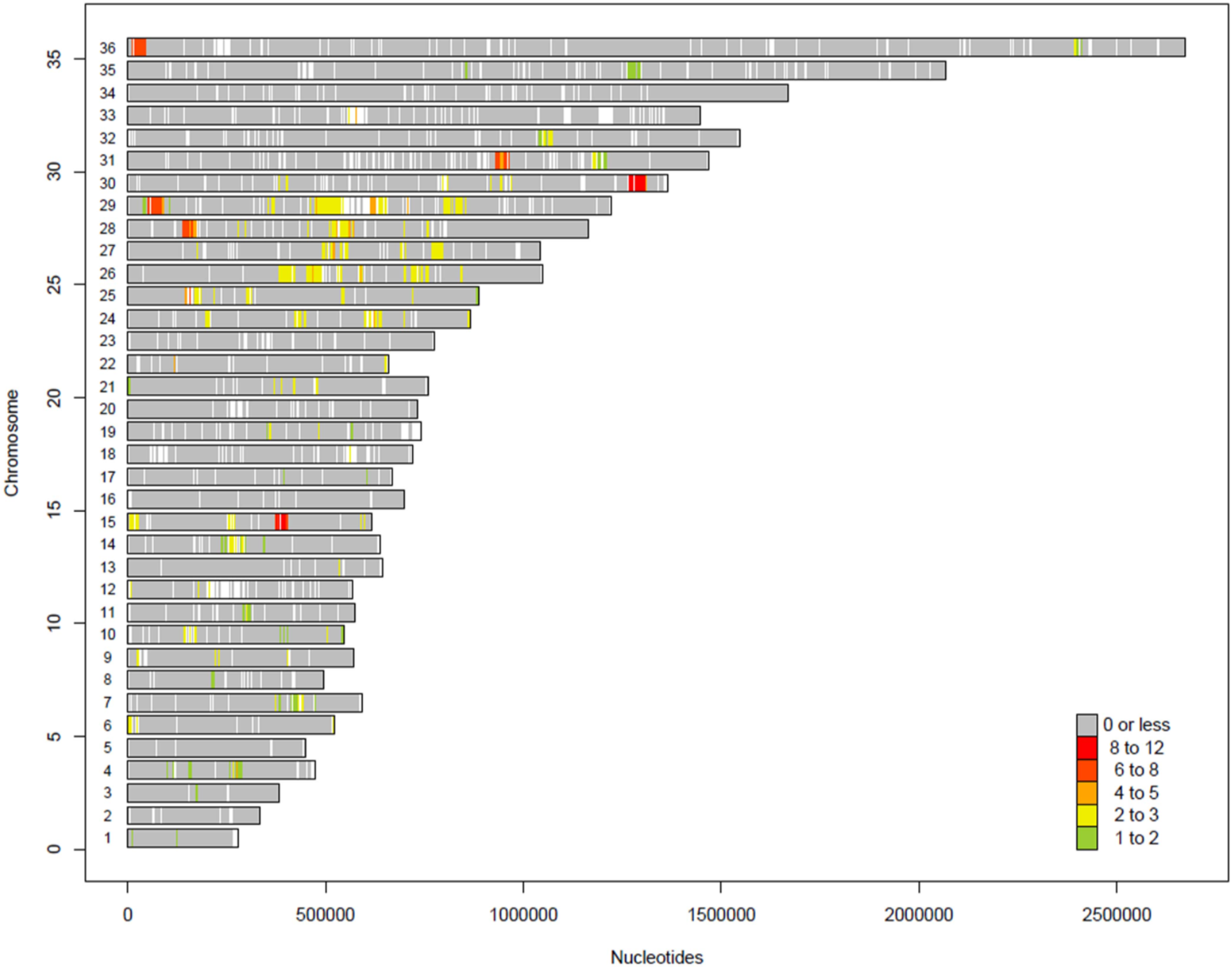

Supplement: FIG S4 [file mSystems.00416-19-sf004.tif]

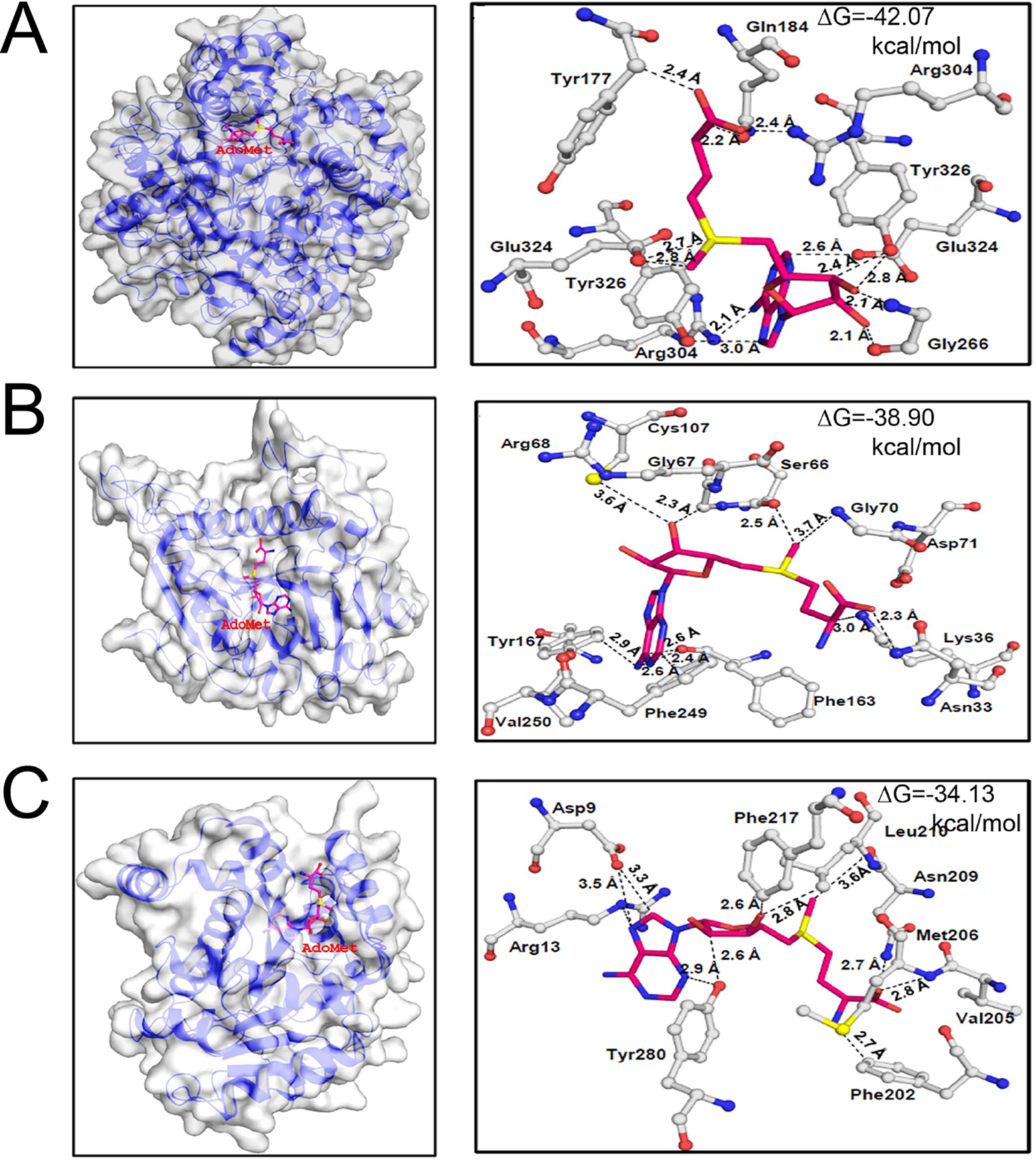

Supplement: FIG S5 [file mSystems.00416-19-sf005.tif]

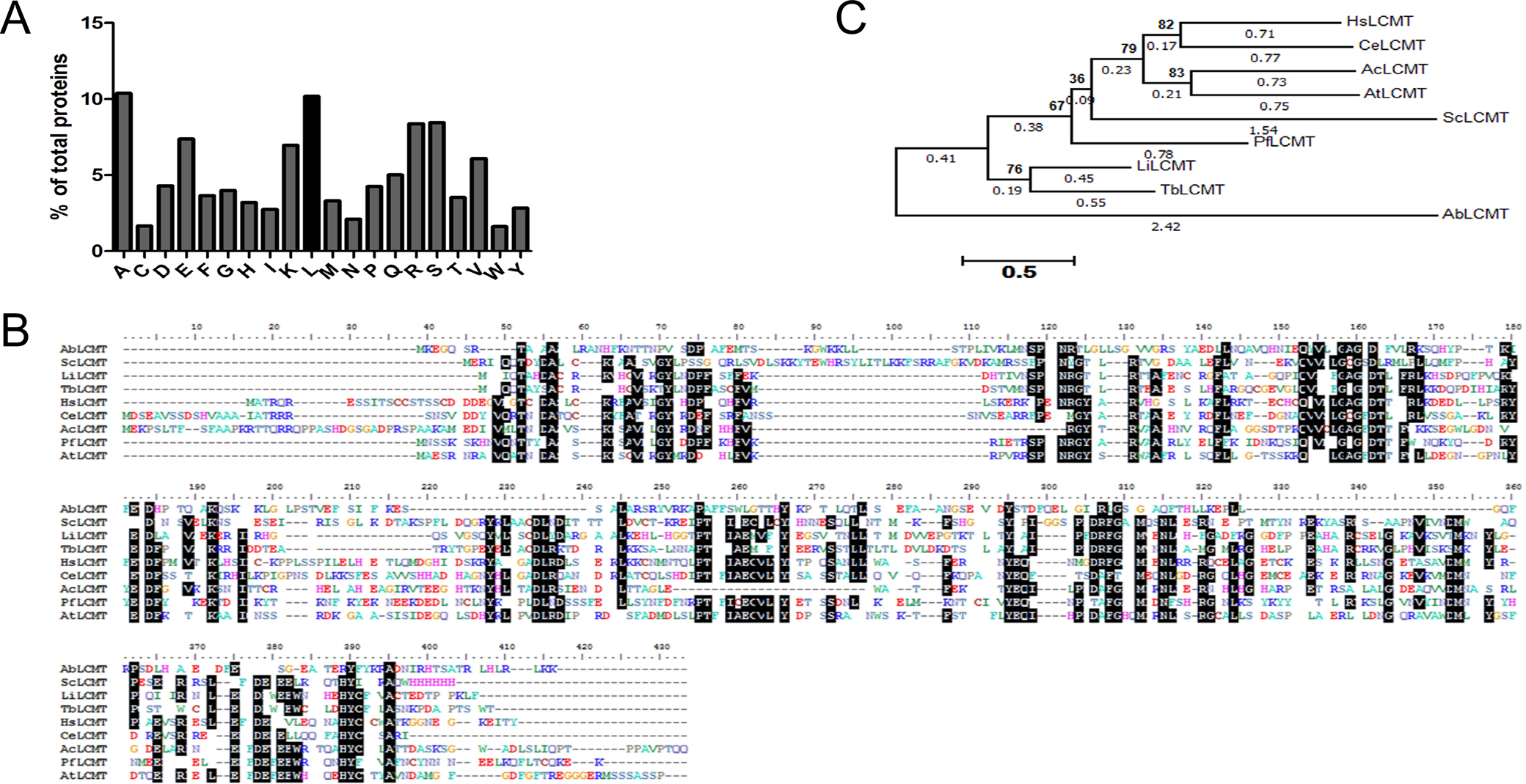

Supplement: FIG S6 [file mSystems.00416-19-sf006.tif]

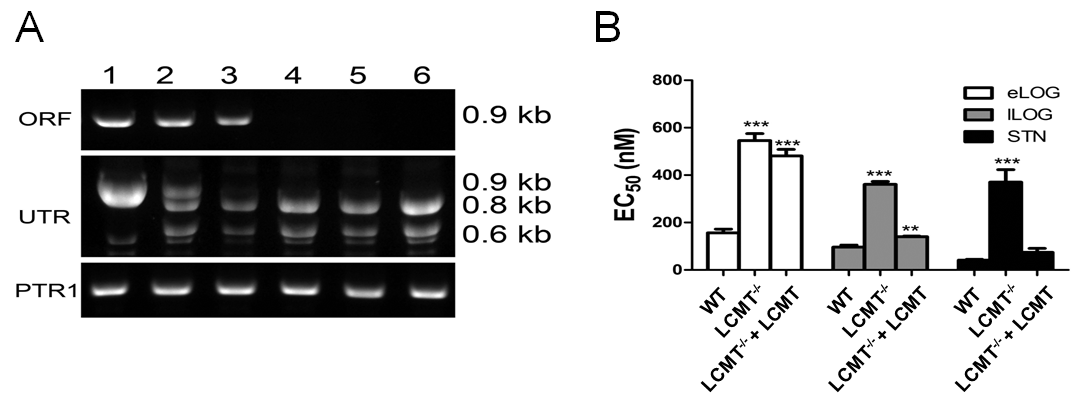

Supplement: FIG S7 [file mSystems.00416-19-sf007.tif]

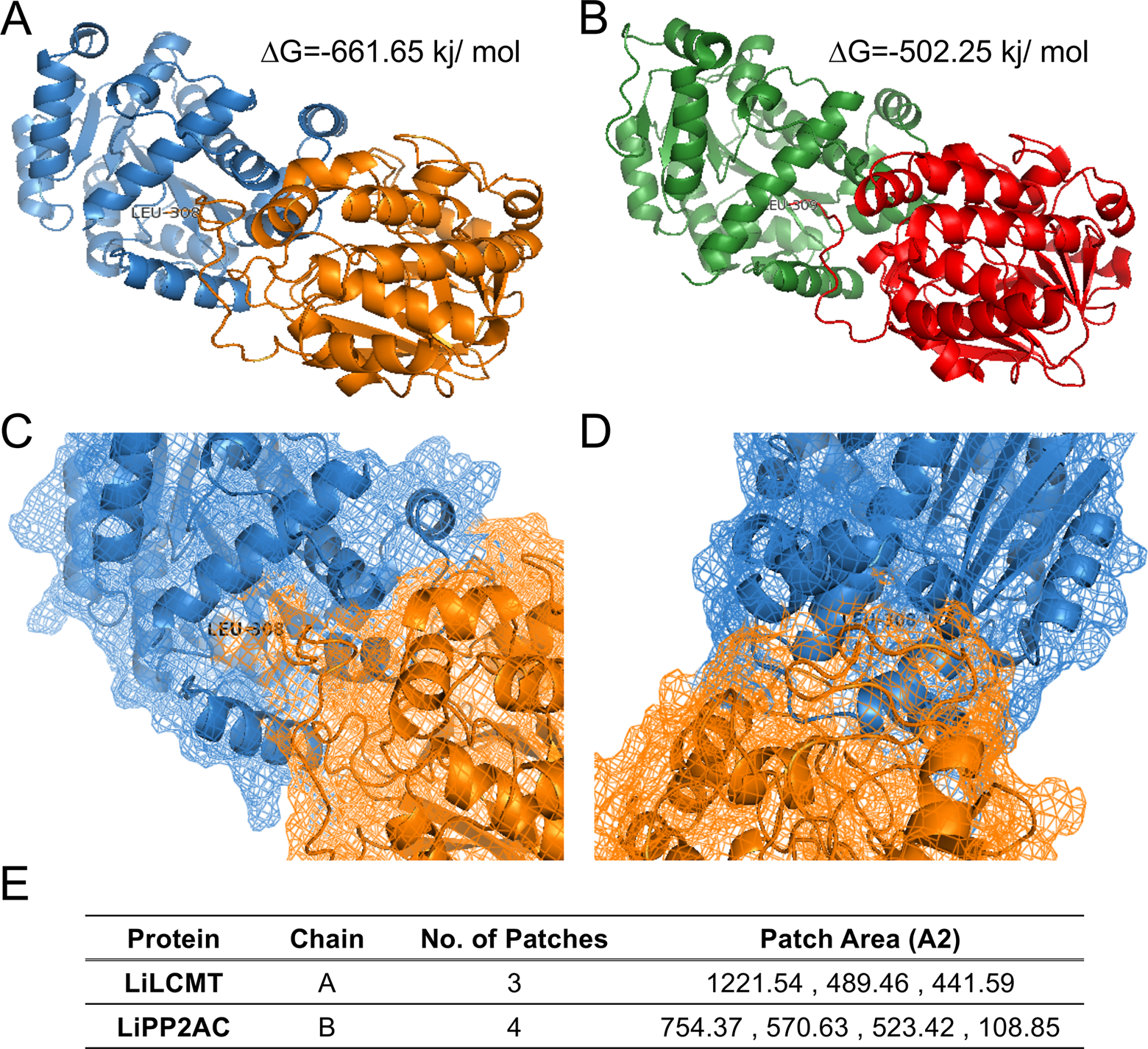

Supplement: FIG S8 [file mSystems.00416-19-sf008.tif]
