# Supplementary material for: Genomewide Analysis of Mode of Action of the S-Adenosylmethionine Analogue Sinefungin in Leishmania infantum
Source: mSystems. 2019 Oct 15;4(5):e00416-19. doi: 10.1128/mSystems.00416-19 (PMC6794121; doi:10.1128/mSystems.00416-19)
Supplement: FIG S3 [file mSystems.00416-19-sf003.pdf]

A

```
LinJ.10.0370      ATGG--------C-GAAGGGTCAGTGTGAGGGCCAAGCCTCTCCCGAGGAGATGAGCGCC  51
LinJ.10.0380      ATGACACGCTCACAAAACCAAGCGCGCACACATGAGCCCTCCCACGACGCCGGGAAGGCG  60
                  ***         *  **      * *        *  **** * *** *    **  **

LinJ.10.0370      GACCCCTACAGAAATGATGCGGCCGTGAAACCGGAAAGGTTTGTGCACCCCGAGGCAGCT  111
LinJ.10.0380      CAGAACAGCGCGCAAGGGTCGCAAGTGACGCAGAAATACTACGTCCATCCGCAGGCAGCC  120
                   *   *  *    * *   **   ****  * * **   *  ** ** **  *******

LinJ.10.0370      GCCTTGTTCTCTAAGTGGCCGTGGGTGCGACGGGTGCCGATGTTTGGCGAGGCTGTCGAG  171
LinJ.10.0380      GCCTTGTTCTCTAAGTGGCCGTGGGTGCGACGGGTGCCGATATTTGGTGAGGCTGTCGAG  180
                  ***************************************** ***** ************

LinJ.10.0370      GGCTACGGGCTCAAGGTCATCGTCGCTCTTGGTGCTACCAACCTGCTCTGCAGAGGCGTC  231
LinJ.10.0380      GGCTACGGGCCCAAGGTCATCGTCGCTCTTGGTACGAGCTACCTGCTCTGCAAGGGCGTC  240
                  ********** ********************** * * * ************  ******

LinJ.10.0370      GCGGATCGTATCCTGACGGGTCAGACGTACGCCATGATGATTGATCGCTACGGCATCGAC  291
LinJ.10.0380      GCGGATCAGATTCTTACCGGTCAGACGTACGCCATGATGATTGATCGCTACGGCATCAGT  300
                  *******  ** ** ** ***************************************

LinJ.10.0370      GTGGCCCGCTACCAGCGCCTTTCCACCATCGCTACGATGGGGTGGTCCATCAAGGCCTTC  351
LinJ.10.0380      GTTCCCCGCTACCAGCGCCTGTCTCCGATTTCGTCCATGGGGTGGTCCATCAAGGCCTTC  360
                  **  **************** **  * **  *  * ************************

LinJ.10.0370      ACAGCGATGCTCTGCGACGGCTTCGCCTTCCTCGGCTACACGAAGCGCTGGTACATGTTC  411
LinJ.10.0380      ACAGCGATGCTCTGCGACGGCTTCGCCTTCCTCGGCTACACGAAGCGCTGGTACATGTTC  420
                  ************************************************************

LinJ.10.0370      ATCTCCTGCGTCGGCGGTGGCGCGTTCGCGCTGATCTACGGCCTCCTTCCGGCGAAGGAG  471
LinJ.10.0380      ATCTCCTGCGTCGGCGGTGGCGCGTTCGCGCTGATCTACGGCCTCCTTCCGGCGAAGGAG  480
                  ************************************************************

LinJ.10.0370      GCGTCGGCTGATGTGGCAGCCGCCTTCATCTTCCTGTCGACGTGGGGCAAGGCCAACGTG  531
LinJ.10.0380      GCGTCGGCTGATGTGGCAGCCGCCTTCATCTTCCTGTCGACGTGGGGCAAGGCCAACGTG  540
                  ************************************************************

LinJ.10.0370      GATATCCTGTCGGAGGGCCATTACAGTCGACTGATGCGCCAGAACCCGAAGCCTGGCCCG  591
LinJ.10.0380      GATATCCTGTCGGAGGGCCATTACAGTCGACTGATGCGCCAGAACCCGAAGCCTGGCCCG  600
                  ************************************************************

LinJ.10.0370      TCCATGGTGAGCTGGATCTGGTTCTGGATCATGGTAGGGGCCATCATCGCGACTGTGATG  651
LinJ.10.0380      TCCATGGTGAGCTGGATCTGGTTCTGGATCATGGTAGGGGCCATCATCGCGACTGTGATG  660
                  ************************************************************

LinJ.10.0370      AACGGCCCGCTCGCGGATGCCGGGAAGCCGCAGATCAGCATCTTCGTGTCTGCCGCGCTG  711
LinJ.10.0380      AACGGCCCGCTCGCGGATGCCGGGAAGCCGCAGATCAGCATCTTCGTGTCTGCCGCGCTG  720
                  ************************************************************

LinJ.10.0370      CAGCTCATCACCTGCGTCTTCTACCTGTTCAACTGGTACGGGGAGAAGAAGAACCGCGTG  771
LinJ.10.0380      CAGCTCATCACCTGCGTCTTCTACCTGTTCAACTGGTACGGGGAGAAGAAGAACCGCGTG  780
                  ************************************************************

LinJ.10.0370      CTGCGTTCCGAGGACGCGCTGTTTATTCTGGAGGAGACGCGCAAGGAGCGTGACCGTTTT  831
LinJ.10.0380      CTGCGTTCCGAGGACGCGCTGTTTATTCTGGAGGAGACGCGCAAGGAGCGTGAGCGCCTG  840
                  ***************************************************** **  *

LinJ.10.0370      AGGCTCCCGGCGCACGATGAACCGGTCACCGGGGTGCCGCAGCATGGTGGTGCGGCGAAG  891
LinJ.10.0380      GGCACTGAACTGAT---GGACGACGGCACGGCGGGTGCGCAGCATGGTGGTGCGGCGAAG  897
                   *         *      **    * *** * **   ***********************

LinJ.10.0370      GGGAAGAGGAGCCCGCAGCGCTCGCACTCGGATGAGGATGTGGAGGCAGTGGCAGTCGAA  951
LinJ.10.0380      GGGAAGAGGAGCCCGCAGCGCTCGCACTCGGATGAGGACGTGGAGGGTGCCGTACGGGAT  957
                  ************************************** *******  *  * *   **

LinJ.10.0370      CAAGTCAATAGAGACGATGTGCACGTCCCGCGCGAGTTGGCGCAGCTACCTTACGAAGGC  1011
LinJ.10.0380      GCCCTCAACGATGGTCAGCGCGACAACGGTGAACTTGTGCAGGACGTCTACGACGACGCG  1017
                      ****    *   *     **  *          **  * *  *     **** *
```

```
LinJ.10.0370      GATGATGACGCGGATGCCGACGCGGCAGCGGAGGTGTACTACGGCAAGCCGCCGGTGCCG  1071
LinJ.10.0380      TATGACGACGGCGAAGAGGTGGCCGAGGGCGAGGTGTACTACGGCAAGCCGCCGGTGCCG  1077
                   **** ****  ** *  *  ** *  *  ******************************

LinJ.10.0370      TGCCTGTTCGGGCTGTTCGAAATGAACAGGGAAGTGATCACAGATAACTGGAAGATCTTC  1131
LinJ.10.0380      TGCCTGTTCGGGCTGTTCGAGGCGAACACGGAGGTGATTTCGAAGAACTGGAAGATCTTC  1137
                  ********************   ***** *** *****  *  * ***************

LinJ.10.0370      GTGTACAGCGTTGTCATGACCTGTGCTGTGATCGCGATGCTGTGTGCCAACATCCTGGCC  1191
LinJ.10.0380      GTGTACAGCGTTGTCATGACCTGTGCTGTGATCGCGATGCTGTGCTGCAACATCCTGGCC  1197
                  ********************************************   *************

LinJ.10.0370      GACACGCTGGGCCTCCTGGTTGCGTGCGTCTTTGTGTCGACCATCTGCTGCTGCTCCTCA  1251
LinJ.10.0380      GACACGCTGGGCCTCCTGATTGCGTGCGTCGTTGTGTCGACCATCTGCTGCGCCACGTCC  1257
                  ****************** *********** ********************  * * **

LinJ.10.0370      TTCTGGGCCCTGCCGCTGGTGATTGCGAAGGCCAACGTCTTTGGCTACCTCCAGCAGGCG  1311
LinJ.10.0380      TTCTGGGCCCTGCCGCTGGTGATTGCGAAGGCGAATGTGTTCTGCTACCTTCAGATGGCC  1317
                  ******************************** ** ** **  ******* ***  ***

LinJ.10.0370      GTGTACATCAACATCGCGAGCCCCCTCATGACCTTCTACCTGAACTCATACAACTGTGAG  1371
LinJ.10.0380      GTCTACATCCGTGCCACTAGTCCACTGTACGCCTTTTATCTGAACTCGAATGAATGCCAA  1377
                  ** ******     * * ** ** **     **** ** ********  *  * **  *

LinJ.10.0370      GAGAACTTCCCGAACTTTAGCTACAGTTTCTACAACACCGTCGCGGGTGTCATCGGCAAC  1431
LinJ.10.0380      GGTGATTACCCCAACTTCACCTACACCTTCTACAACACGGTGGCTGGCGTGATTGGCAAC  1437
                  *   * * *** ***** * *****  *********** ** ** ** ** ** ******

LinJ.10.0370      TTCGCAGGGCTAGCTGGCGTGTCCGCATTCAACTGCATATTCTCGAAGCGCAGCTACCGC  1491
LinJ.10.0380      CTCGCAGGGCTGGTCGGCGTGACGCTGTTCAACTTCCTGTTCGCGAAGCATAACTATCAG  1497
                   ********** *  ****** *    ******* * * *** ******  * *** *

LinJ.10.0370      CTCACCTTCTGCGTGACCACGTTCGCGCAGGTTCTGGGCGGAATGACGGATATTGTGATT  1551
LinJ.10.0380      GTGACCTTCATTGTGACGACAATCATGCAAGTTCTGGCAGCGCTGTTCGACATCATCATG  1557
                   * ******   ***** **  **  *** *******  *   **   ** **  * **

LinJ.10.0370      GTGAAGCGCTGGAACCTGTACATCGGCATCCCTGACCACGCCATGTACATCTGGGGTGCG  1611
LinJ.10.0380      GTGAAGCGCTGGAACAAGCGCATCGGCATCCCTGACCATGCCATGTACATCTGGGGTGAT  1617
                  ***************  *  ****************** *******************

LinJ.10.0370      GCTGTAGTGAGCGAGGTGTGCTACATGCTTGGCTATATGCCGATGGTTGTGCTGCTGTCT  1671
LinJ.10.0380      GCGGTTGTAGCACAGATTGTTTACATGCTTGGTTTCATGCCGTTGGTTGTGATGCTGTCT  1677
                  ** ** **     ** *    *********** *  ****** ******** ********

LinJ.10.0370      CGCCTGTGCCCTCGTGGCTCGGAGAGTGTCGTGTATGCGCTGATGGCGGGCTTCGCGAGC  1731
LinJ.10.0380      CGCCTGTGCCCTCGTGGCTCGGAGAGTGTCGTGTATGCGCTGATGGCGGGCTTCGCGAAT  1737
                  **********************************************************

LinJ.10.0370      CTGGGACGCTCGACTTCCGCGTCTCTCGGTGCGATCATCATGGAGTACGGCCTGCCTGTG  1791
LinJ.10.0380      CTTGGCCAGACCACCTCGTCGTCCCTCGCTGCGATCATCATGGAGTACGGCTGGCCTATT  1797
                  ** ** *   * ** **  **** **** **********************  **** *

LinJ.10.0370      TTCAAGACGCGGGACGATGGCTACCGTTGCGGCGTGGAGAATCTTGCATGGCTGCTGTTT  1851
LinJ.10.0380      TTTTCGGATAACGACCCATGC---------AACTACGACAACCTGCCGCTGCTGCTGTTC  1848
                  **   *      ***    **           *   ** ** **  *   *********

LinJ.10.0370      GTGTGCAATGTGTGTGCGCCTCCGCTCGTGCTACCGCTGACTCTGCTGCTGCCGAAGGCG  1911
LinJ.10.0380      GTGACCAGCGTCTGCACGCCGCTGCTGGTGATTCCACTTTCGTACTTGCTCCCGATGGCG  1908
                  ***  **  ** **  **** * *** *** * ** **  *     **** **** ****

LinJ.10.0370      CGCATCTGCGACGATATCGACATTGACGGCAAGGCGTTGCGCAGGAAGGTAGACGCGGAG  1971
LinJ.10.0380      CGAATCATTGATGATGTGGACATTGACGGCAAGGTGGTGCGTCAGAAGGCTGATGAGATG  1968
                  ** ***   ** *** * **************** * ****   *****  ** * *  *

LinJ.10.0370      TTGATGGCGGGCGAGGCAGGCGATT------TGCCGTCGCCGCCCTCGTCGGCTGAGAAC  2025
LinJ.10.0380      CGCGCGAAGGTAATGGCCGCAGCCAAGGACAGCACCTTGGAGCACTCCGCGGAGGCTTCG  2028
                       *  **    *** *  *            * * *  ** ***  ***  *

LinJ.10.0370      CCCCGTGCGACGAAGGCACAAGATGACGAGGCGACGCGAGAGAAGGTCTAG   2076
LinJ.10.0380      GCC---GCGGCTGCTGCGCCAAAGGG-----CCACTT----------GTAA   2061
                   **   *** *    ** * * * *      * **             **
```

B

ATGGCGAAGGGTCAGTGTGAGGGCCAAGCCTCTCCCGAGGAGATGAGCGCCGACCCCTA
CAGAAATGATGCGGCCGTGAAACCGGAAAGGTTTGTGCACCCCGAGGCAGCTGCCTTGT
TCTCTAAGTGGCCGTGGGTGCGACGGGTGCCGATATTTGGTGAGGCTGTCGAGGGCTAC
GGGCCCAAGGTCATCGTCGCTCTTGGTACNAGCTACCTGCTCTGCAAGGGCGTCGCGGA
TCAGATTCTTACCGGTCAGACGTACGCCATGATGATTGATCGCTACGGCATCAGTGTTC
CCCGCTACCAGCGCCTGTCTCCGATTTCGTCCATGGGGTGGTCCATCAAGGCCTTCACA
GCGATGCTCTGCGACGGCTTCGCCTTCCTCGGCTACACRAAGCGCTGGTACATGTTCAT
CTCCTGCGTCGGCGGTGGCGCGTTCGCGCTGATCTACGGCCTCCTTCCGGCGAAGGAGG
CGTCGGCTGATGTGGCAGCCGCCTTCATCTTCCTGTCGACGTGGGGCAAGGCCAACGTG
GATATCCTGTCGGAGGGCCATTACAGTCGACTGATGCGCCAGAACCCGAAGCCTGGCCC
GTCCATGGTGAGCTGGATCTGGTTCTGGATCATGGTAGGGGCCATCATCGCGACTGTGA
TGAACGGCCCGCTCGCGGATGCCGGGgAAGCCGCAGATCAGCATCTTCGTGTCTGCCGC
GCTGCAGCTCATCACCTGCGTCTTCTACCTGTTCAACTGGTACGGGGAGAAgAAGAACC
GCGTGCTGCGTTCCGAGGACGCGCTGTTTATTCTGGAGGAGACGCGCAAGGAGCGTGAG
CGCCTGGGCACTGAACTGATGGACGACGGCACGGCGGGTGCGCAGCATGGTGGTGCGGC
GAAGGGGAAGAGGAGCCCGCAGCGCTCGCACTCGGATGAGGACGTGGAGGGTGCCGTAC
GGGATGCCCTCAACGATGGTCAGCGCGACAACGGTGAACTTGTGCAGGACGTCTACGAC
GACGCGTATGACGACGGCGAAGAGGTGGCCGAGGGCGAGGTGTACTACGGCAAGCCGCC
GGTGCCGTGCCTGTTCGGGCTGTTCGAGGCGAACACGGAGGTGATTTCNAAGAACTGGA
AGATCTTCGTGTACAGCGTTGTCATGACCTGTGCTGTGATCGCGATGCTGTGCTGCAAC
ATCCTGGCCGACACGCTGGGCCTCCTGATTGCGTGCGTCGTTGTGTCGACCATCTGCTG
CGCCACGTCCTTCTGGGCCCTGCCGCTGGTGATTGCGAaGGCGAATGTGTTCTGCTACC
TTCAGATGGCCGTCTACATCCGTGCCACTAGTCCACTGTACGCCTTTTATCTGAACTCG
AATGAATGCCAAGGTGATTACCCCAACTTCACCTACACCTTCTACAACACGGTGGCTGG
CGTGATTGGCAACCTCGCAGGGCTGGTCGGCGTGACGCTGTTCAACTTCCTGTTCGCGA
AGCATAACTATCAGGTGACCTTCATTGTGACGACnAATCATGCAAGTTCTGGCAGCGCT
GTTCGACATCATCATGGTGAAGCGCTGGAACAAGCGCATCGGCATCCCTGACCATGCCA
TGTACATCTGGGGTGATGCGGTTGTAGCACAGATTGTTTACATGCTTGGTTTCATGCCG
TTGGTTGTGATGCTGTCTCGCCTGTGCCCTCGTGGCTCGGAGAGTGTCGTGTATGCGCT
GATGGCGGGCTTCGCGAATCTTGGCCAGACCACCTCGTCGTCCCTCGCTGCGATCATCA
TGGAGTACGGCTGGCCTATTTTTTCGGATAACGACCCATGCAACTACGACAACCTGCCG
CTGCTGCTGTTCGTGACCAGCGTCTGCACGCCGCTGCTGGTGATTCCACTTTCGTACTT
GCTCCCGATGGCGCGAATCATTGATGATGTGGACATTGACGGCAAGGTGGTGCGTCAGA
AGGCTGAWnGAGATGCGCGCGAAGGTAATGGCCGCAGCCAAGGACAGCACCTTGGAGCA
CTCCGCGGAGGCTTCGGCCGCGGCTGCTGCGCCAAAGGGCCACTTGTAA
