# Supplementary material for: Genomewide Analysis of Mode of Action of the S-Adenosylmethionine Analogue Sinefungin in Leishmania infantum
Source: mSystems. 2019 Oct 15;4(5):e00416-19. doi: 10.1128/mSystems.00416-19 (PMC6794121; doi:10.1128/mSystems.00416-19)
Supplement: TABLE S1 [file mSystems.00416-19-st001.pdf]

Number of mutants with gene mutated	Mutants ID	Gene ID	Product
4	50.1, 50.2, 50.3, 50.4	LinJ.35.0520	Proteophosphoglycan
		LinJ.35.0530	Proteophosphoglycan
3	50.1, 50.2, 50.3	LinJ.19.1680	Hypothetical protein
	50.1, 50.3, 50.4	LinJ.19.1690	Hypothetical protein
	50.1, 50.2, 50.3	LinJ.35.0500	Proteophosphoglycan
2	50.1, 50.4	LinJ.08.0950	Cathepsin L-like protease
2	50.3, 50.4	LinJ.14.1180	Kinesin
2	50.2, 50.4	LinJ.26.2180	Hypothetical protein
2	50.1, 50.4	LinJ.31.3330	Proteophosphoglycan
2	50.2, 50.4	LinJ.35.0490	Proteophosphoglycan
2	50.1, 50.3	LinJ.35.0510	Proteophosphoglycan
